# An herbal formula attenuates collagen-induced arthritis via inhibition of JAK2-STAT3 signaling and regulation of Th17 cells in mice

**DOI:** 10.18632/oncotarget.17797

**Published:** 2017-05-11

**Authors:** Xin Wu, Qiyang Shou, Cuiwei Chen, Hao Cai, Jida Zhang, Songqi Tang, Baochang Cai, Dongxin Tang, Gang Cao

**Affiliations:** ^1^ Research Center of TCM Processing Technology, Zhejiang Chinese Medical University, Hangzhou, China; ^2^ Experimental Animal Research Center, Zhejiang Chinese Medical University, Hangzhou, China; ^3^ School of Pharmacy, Zhejiang Chinese Medical University, Hangzhou, China; ^4^ Department of Traditional Chinese Medicine, Zhejiang Cancer Hospital, Hangzhou, China; ^5^ School of Pharmacy, Nanjing University of Chinese Medicine, Nanjing, China; ^6^ College of Basic Medical Science, Zhejiang Chinese Medical University, Hangzhou, China; ^7^ Hangzhou Herbal Chinese Medicine Co., Ltd, Hangzhou, China; ^8^ College of TCM, Hainan Medical University, Haikou, China; ^9^ Guiyang University of Chinese Medicine, Guiyang, China

**Keywords:** JAK2-STAT3 pathway, Th17 cells, rheumatoid arthritis, wenjinghuoluo prescription, herbal formula

## Abstract

Wenjinghuoluo prescription, a traditional Chinese medicine compound treatment of rheumatoid arthritis characterized by wind–cold–dampness arthralgia, contains five herbs, namely, *C. cassia* Presl., *Cinnamomum cassia* Presl., *Paeonia lactiflora* Pall., *Saposhnikovia divaricate* (Turcz.) Schischk., and *Clematis chinensis* Osbeck. We have reported that WJHL could inhibit the production of inflammatory mediators in immune cells. This study explored the effect and mechanism of WJHL on collagen-induced arthritis mice. WJHL could significantly improve clinical arthritic conditions; inhibit bone erosion and osteophyte formation in joints; decrease expression of proinflammatory cytokines (TNF-α, IL-1β, IL-6, and IL-17); reduce protein expression levels of JAK2, p-JAK2, STAT3, p-STAT3 and gene expression levels of JAK2, STAT3, IL-17A, RORγt mRNA; elevate osteoprotegerin and Foxp3 mRNA levels and lower Th17 cell proportions in splenocytes. Results suggest that WJHL, specifically regulating the JAK2/STAT3 pathway and Th17 cells, may be a promising herbal medicine candidate for the treatment of RA.

## INTRODUCTION

Rheumatoid arthritis (RA) is a type of chronic systemic inflammatory disease with disorders of immune regulation [1]. The main clinical manifestations of RA include multi-joint symmetric, invasive arthritis, and *extra*-*articular* organ involvement [2]. Some patients present with fever, fatigue, pleuritis, pericarditis, arteritis, subcutaneous nodules, peripheral neuropathy, major depressive disorder, and other physical and mental diseases [3]. RA usually causes severe disability or even premature death [4]. A longer course of RA and the lack of effective treatment result in increased cost of treatment, leading to considerable financial burden to the patients and their families across the country [5]. How to effectively prevent and treat RA has now become a major issue that should be solved by the pharmaceutical industry.

The pathogenesis of RA is complex, and the activation and development of RA autoimmune response are presumed to be caused by the autoimmune response of T cells to the antigenic polypeptide presented by dendritic cells (DCs) and other antigen-presenting cells (APCs) [6]. T cell-mediated immune abnormality is the main pathogenetic pathway of RA [7]. In particular, CD4^+^ T cells can trigger and prolong inflammation [8]. T cell differentiation in the inflammatory site is mediated by the Janus tyrosine kinase (JAK)/signal transducer and activator of transcription (STAT) signaling pathway [9]. Activated CD4^+^ T cells are differentiated into Th17 cells by activation of the IL-6-activated STAT3 pathway, and Th17 cells are a subset of CD4^+^ T cells that can secrete IL-17 [10]. Moreover, in the study of the negative regulation of Th17 cell differentiation, cytokine signal transduction SOCS3 is an important negative regulation factor, which acts by inhibiting the phosphorylation of STAT3 [11]. RORγt is presumed to be a transcriptional activator specific for Th17 cell lineages that can induce a high level of secretion of IL-17 [12]. The previously presented evidence confirms that Th17 and its effector IL-17 have key pathogenic effects in all stages of RA development (i.e., synovial inflammation, cartilage destruction, and bone destruction). SiRNA or the JAK2/STAT3 pathway inhibitor of STAT3 can inhibit Th17 cell differentiation, inhibit osteoclast function, and ease the pain of collagen-induced arthritis (CIA) in mice [13]. Therefore, the inhibition of the activation of the JAK2/STAT3 signaling pathway and the regulation of Th17 differentiation will be important strategies for the treatment of RA.

The wenjinghuoluo prescription (WJHL) is the traditional Chinese medicine treatment of RA characterized by wind–cold–dampness *arthralgia*. The drug prescription contains *C. cassia* Presl., *Cinnamomum cassia* Presl., *Paeonia lactiflora* Pall., and two other ingredients of *Saposhnikovia divaricate* (Turcz.) Schischk. and *Clematis chinensis* Osbeck., which can dispel wind, remove obstruction in the meridians, and maintain the balance of *yin* and *yang* [14]. *C. cassia* Presl. and *Paeonia lactiflora* Pall. comprise the monarch drugs in the prescription. *Clematis chinensis* Osbeck. can also dispel wind, remove obstruction in the meridians, eliminate dampness, relieve pain, and enable the *recuperation of* five organs [15]. Thus, *Clematis chinensis* Osbeck. is called ministerial drug. *Saposhnikovia divaricate* (Turcz.) Schischk. is the nourishing agent among the disease-modifying antirheumatic drugs; it can warm meridians, relieve pain, and eliminate wind–dampness [16]. *Saposhnikovia divaricate* (Turcz.) Schischk. comprises the adjuvant drug in the prescription. *Cinnamomum cassia* Presl. can work together with *C. cassia* Presl. to invigorate the pulse, ease pain, warm meridians, and enhance *yang*. Thus, *Cinnamomum cassia* Presl. is called assistant drug and adjuvant drug. Together, the five kinds of chinese medicine can ease pain, relieve the symptoms of patients, lower body temperature, expel wind, warm meridians, dispel pathogenic cold, and resolve dampness, which can result in a permanent cure for RA, reduce the morbidity of the disease, and improve patient survival and living skills. In this study, DBA/1 mice are used to set up the CIA model [17]. WJHL was given to mice via intragastric administration. The therapeutic effect of WJHL on RA was further clarified by the pathological changes of arthritis swelling, arthritis index, and joint tissue. Thus, this study explores the effect of WJHL on the JAK2/STAT3 signaling pathway and Th17 differentiation in the CIA mice to reveal the mechanism of WJHL therapy for RA. Hopefully, it could be a new immunization target drug for the treatment of RA.

## RESULTS

### WJHL improved clinical arthritic conditions in CIA mice

**Figure 1:**
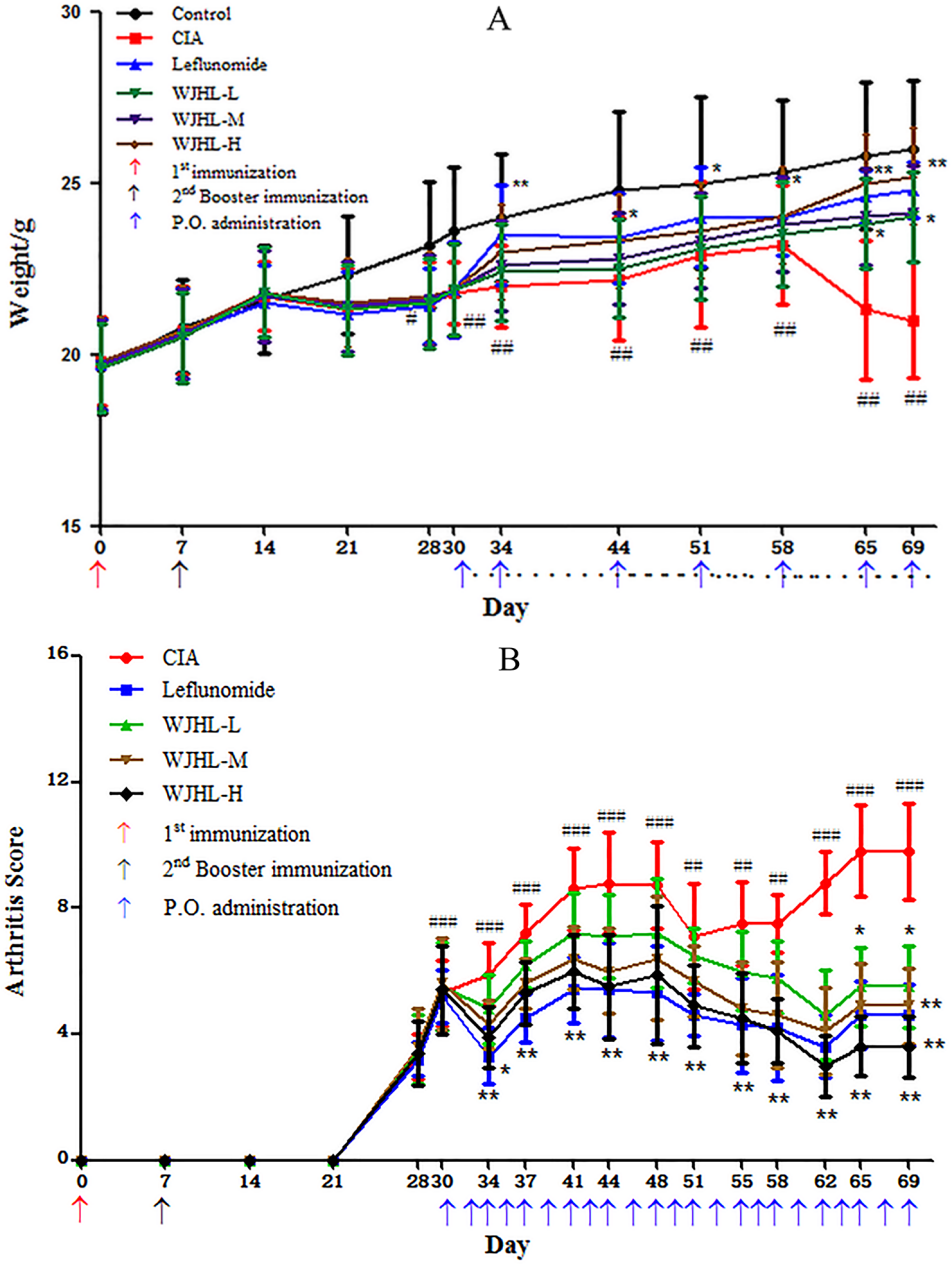
Effects of WJHL on disease progression in CIA mice Values are the mean ± SEM (*n* = 10). *P < 0.05 and ^*^P < 0.01 compared with the CIA group; ^#^*P <* 0.05, ^##^*P <* 0.01, and ^###^*P <* 0.001 compared with the control group. (**A**) Weight changes. (**B**) Arthritis score.

Experimental observation shows that the control group of mice is in good condition with shiny hair, increased weight, normal diet, and flexible response. Figure 1A shows that the weight of the normal control group of mice increased steadily. Compared with the normal control group, on the 14th day of feeding, the mice in the CIA group and other treatment groups were not in good condition with withered hair and decreased food intake. Moreover, their weight initially decreased obviously and then increased slowly. The body weight of the leflunomide group is the heaviest in the treatment group on the 34th day, which is significantly different from that of the CIA group (*P < 0.01*). However, on the 58th day, the body weight of the high-dose WJHL group significantly increased and gradually surpassed that of the leflunomide treatment group. On the 69th day, the weight of the high-dose WJHL group is higher than that of the leflunomide group, which is significantly different from that of the CIA group (*P < 0.01*). These results indicate that each dose group of WJHL has the effect of alleviating the weight loss of mice.

Figure 1B shows that on the 30th day of immunization, the CIA exhibited a high statistical difference compared with the control group (*P < 0.001*). The pathological means of detection indicate that the CIA model is set up successfully. Compared with the CIA group, the severity of arthritis in mice in the WJHL group and leflunomide group was alleviated after the 30th day. On the 34th day of immunization, RA symptoms were briefly reduced (*P < 0.01*). On the 44th day of immunization, RA developed to the first peak, and the difference between the CIA and control groups is obvious (*P < 0.001*). On the 58th day of immunization, the arthritis score of the high-dose WJHL group significantly decreased, indicating that WJHL has a certain effect on RA in mice (*P < 0.01*). On the 69th day of immunization, the difference between the CIA and control groups is significant (*P < 0.001*). The high-dose and medium-dose WJHL groups are significantly different from that of the CIA group (*P < 0.01*), and an obvious difference was observed between the low-dose WJHL and CIA groups (*P < 0.05*). From the arthritis score index, the WJHL group exhibited a certain effect on RA at different stages of morbidity, particularly in the late phase of CIA treatment.

### Effect of WJHL on the main organ index of CIA mice

**Figure 2:**
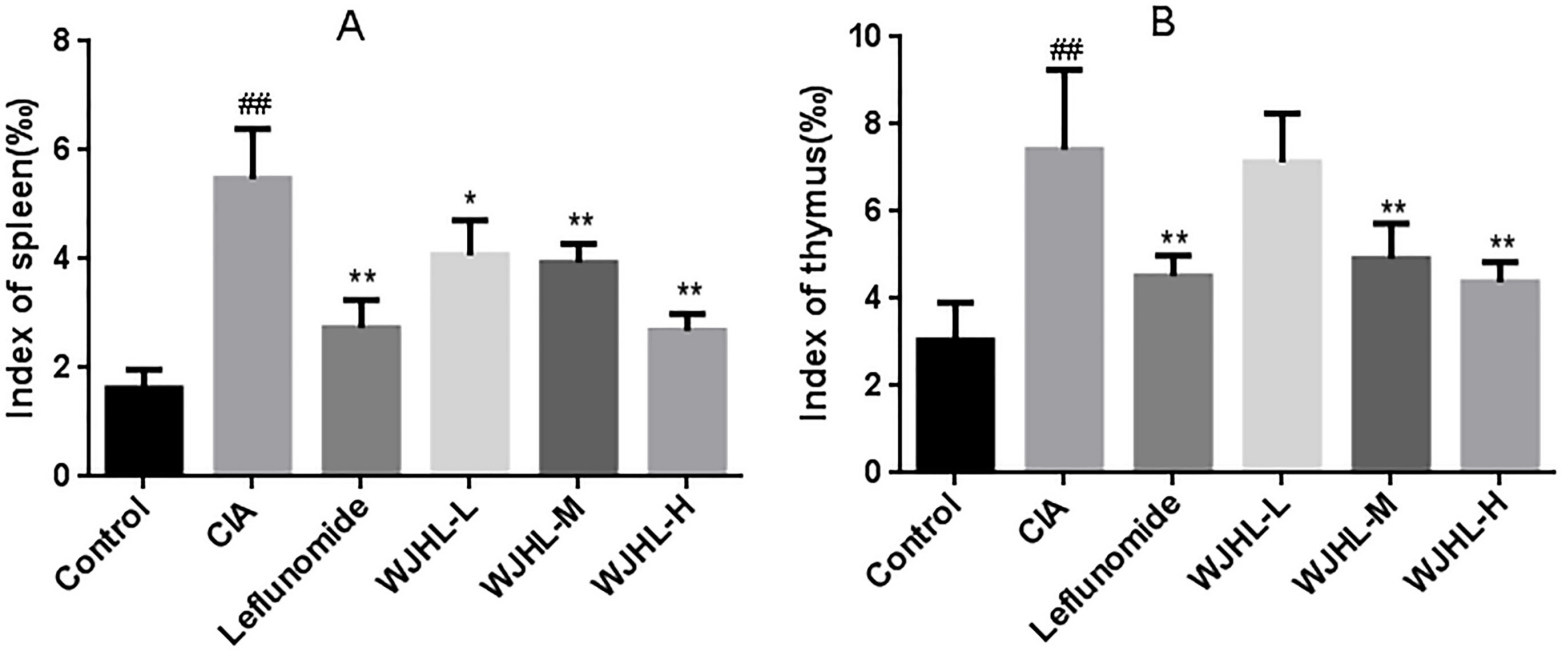
Effect of WJHL on the main organ index of CIA mice Values are the mean ± SEM (*n* = 10). *P < 0.05, ^*^P < 0.01 compared with the CIA group; ^#^*P <* 0.05, ^##^*P <* 0.01 compared with the control group. (**A**) Index of spleen. (**B**) Index of thymus.

The thymus and spleen are the main immune organs of the human body. The organ indices of the thymus and spleen can reflect the development of immune organs and the function of immune cells and indirectly reflect the immune level of the body. Figure 2A and 2B) shows that, compared with the normal mice, the spleen and thymus of the CIA mice were swollen, their weight increased significantly, and the indices of their thymus and spleen increased significantly. The medium-dose and high-dose WJHL groups and leflunomide-positive group of CIA mice exhibited significantly reduced weights and indices of the thymus and spleen (*P < 0.01*). These results are significant, and the inhibition of the high-dose WJHL group on the indices of the thymus and spleen of CIA mice is slightly better than that of the leflunomide-positive group.

### Effect of WJHL on the pathology of ankle tissue of CIA mice

**Figure 3:**
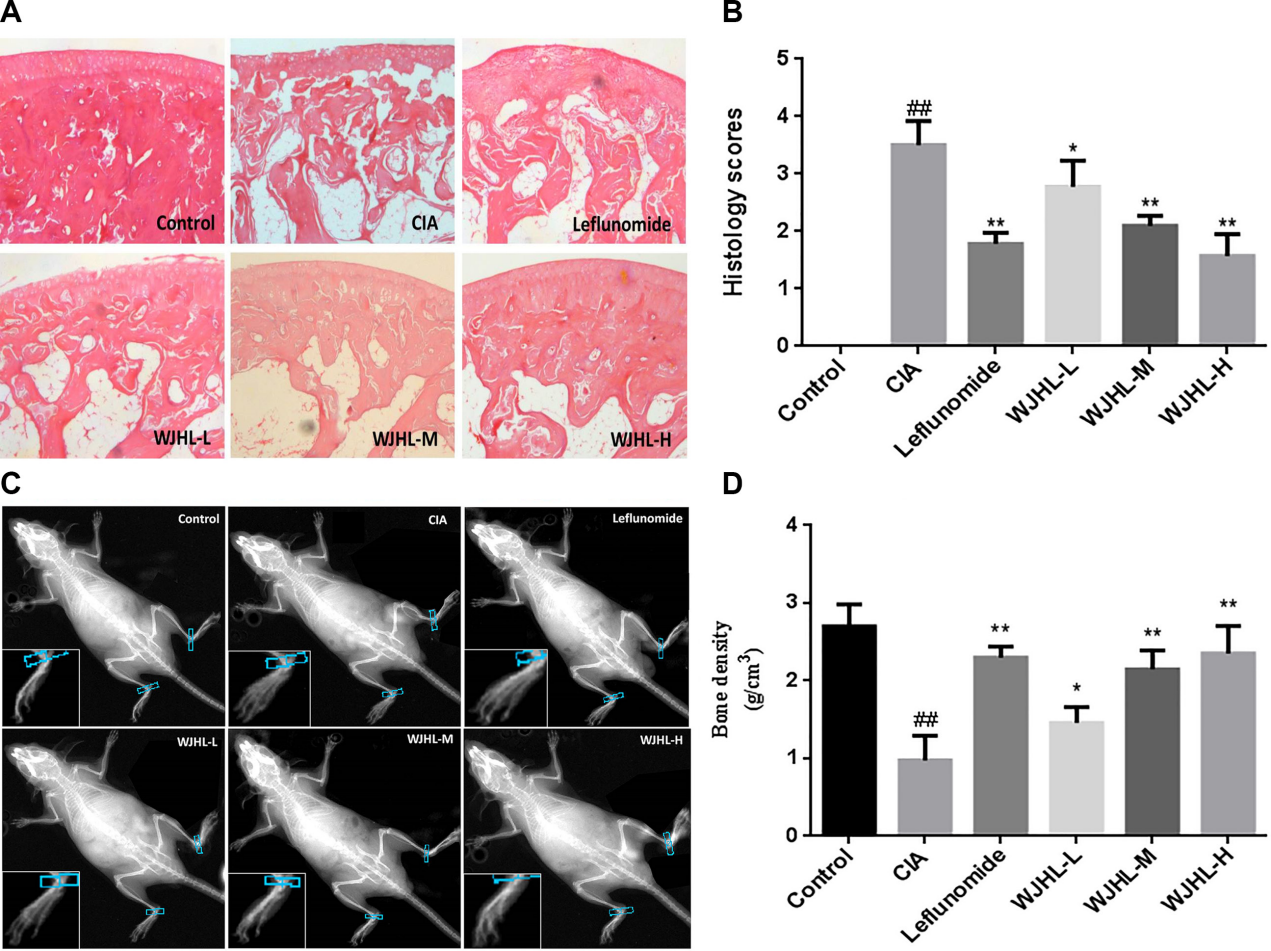
Effect of WJHL on the pathology of ankle tissue of CIA mice Values are the mean ± SEM (*n* = 10). *P < 0.05, ^*^P < 0.01 compared with the CIA group; ^#^*P <* 0.05, ^##^*P <* 0.01 compared with the control group. (**A**) Soft tissue and structure of the ankle of CIA mice by H&E staining. (**B**) Histology score. (**C**) Joint damage by X-ray imaging. (**D**) Bone density.

H&E staining Figure 3A shows that the soft tissue and the structure of the ankle of mice in the control group are normal; the cells are arranged neatly; the surface of ankle cartilage is smooth; the synovial tissue structure is undamaged; no congestion on synovialis is observed; and no significant inflammatory cell infiltration and neovascularization are observed. However, in the CIA group, synovial tissue is hyperplastic and disordered; articular cavity is narrow; significant inflammation and considerable inflammation cell infiltration are observed; and synovium is accompanied by synovial hyperplasia and the formation of neovascularization and pannus. Among all of the treatment groups, synovial cell proliferation is alleviated; congestion and edema of synovial tissue are significantly reduced; the number of infiltrated inflammatory cells is significantly decreased, and bone or cartilage damage is significantly reduced. The grading *results of histology scores* show that, compared with the CIA group, the inflammatory cell infiltration degree in the low-dose, medium-dose, and high-dose WJHL groups decreases by 20.90 ± 3.54%, 39.85 ± 2.45%, and 55.48 ± 5.52%, respectively Figure 3B. The effect of pre-administration *and post*-administration on joint swelling and destruction in CIA mice is observed by X-ray imaging. The results show that, in the control group, joint soft tissue is not swollen and the joint is undamaged. In the CIA group, joint soft tissue is swollen, joint structure is damaged seriously, and bone density decreases more significantly than that in the control group. However, joint damage and soft tissue swelling are significantly relieved, and bone density is significantly high in the WJHL groups after administration of the drug. The bone density result of the high-dose WJHL group is similar to that of the leflunomide-positive group Figure 3C and 3D.

### Effect of WJHL on the treatment of joint injury in CIA mice tested by MICRO-CT

**Figure 4:**
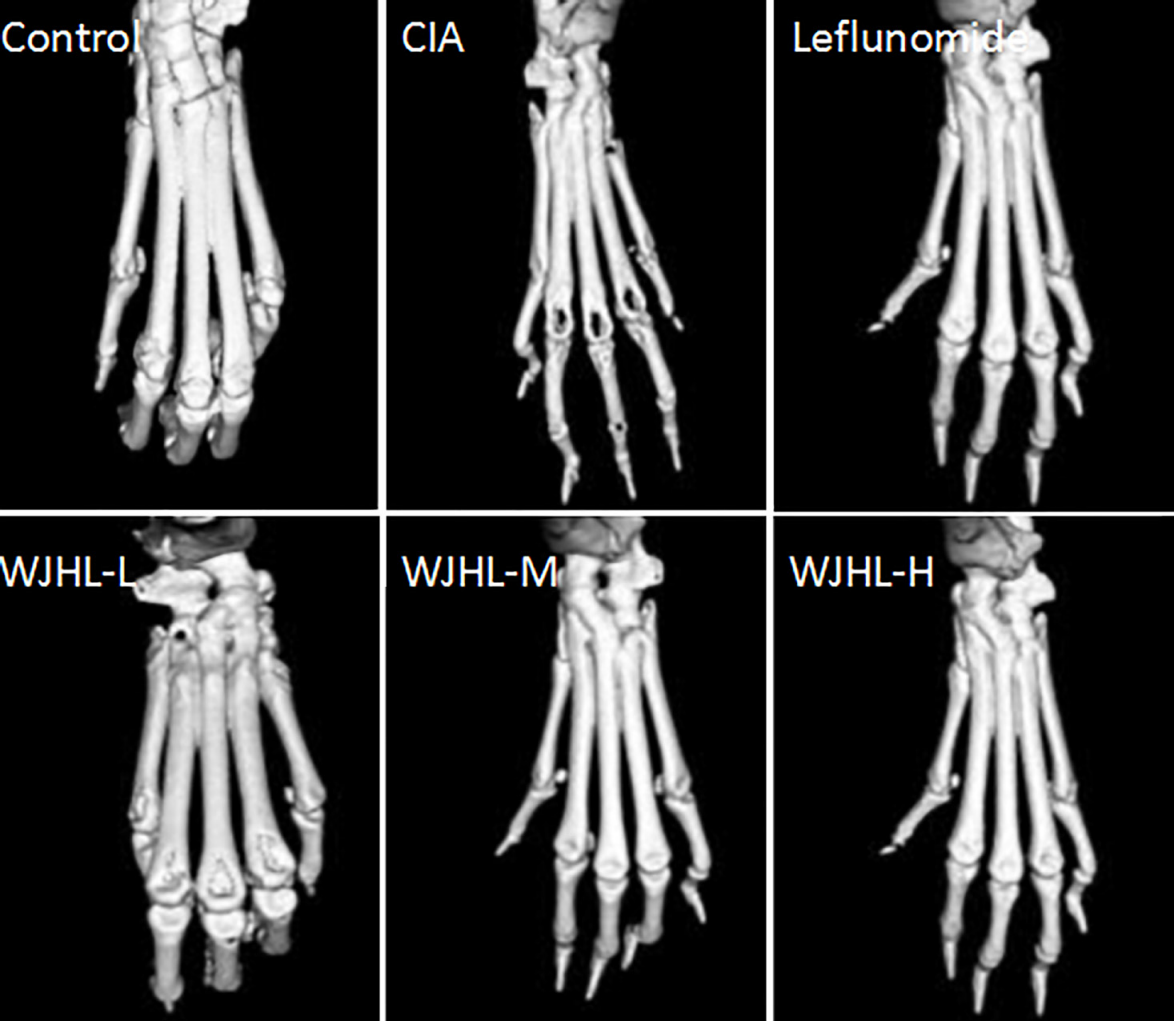
Effect of WJHL on the treatment of joint injury in CIA mice tested by MICRO-CT

After the mice in all groups are killed, their ankle joints and surrounding tissues are fixed with 4% paraformaldehyde and analyzed through *in vitro* MICRO-CT dedicated to small animals. The results shown in Figure 4 indicate that the ankles and surrounding tissues of mice in the control group are not wounded and have no abnormal changes. By contrast, the ankles of CIA mice are seriously damaged, claw joints are eroded to the extent that holes are generated, the shape of the feet is disordered, joint cavities are narrowed, bone is seriously injured, and surfaces of cartilage tissues are rough. The conditions of the medium-dose and high-dose WJHL groups are significantly improved compared with the CIA group, maintaining an intact joint structure and normal joint space. This finding indicates that the severity of bone injury of CIA mice is significantly reduced and the arthritis is alleviated to a certain extent after the administration of WJHL, which prevents the damage of joint tissues and restores the shapes of feet.

### Effect of WJHL on the expressions of JAK2, p-JAK2, STAT3, and p-STAT3 in the spleen of CIA mice tested by immunohistochemistry

**Figure 5:**
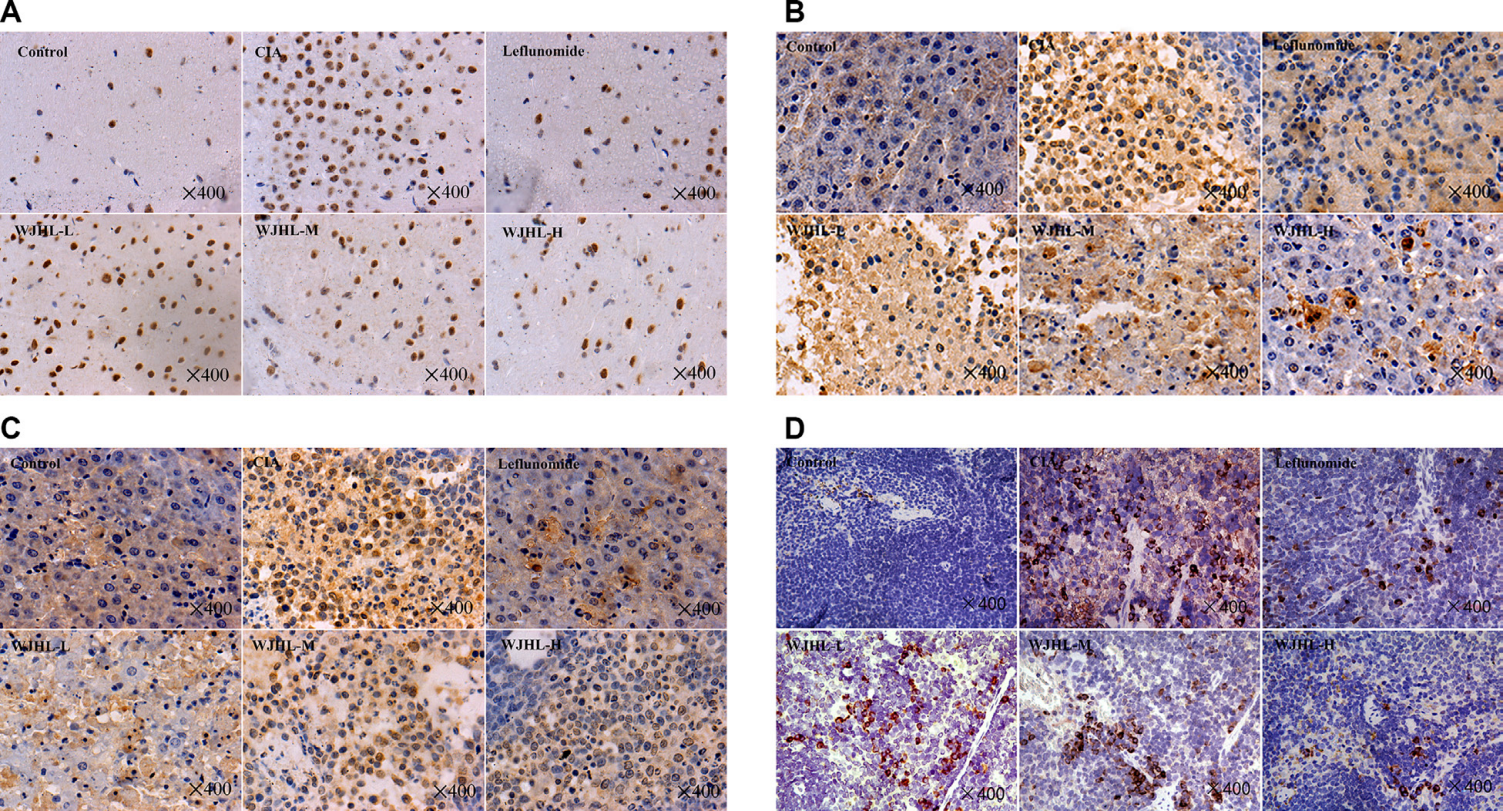
Effect of WJHL on the expressions of JAK2, p-JAK2, STAT3, and p-STAT3 in the spleen of CIA mice tested by immunohistochemistry (**A**) JAK2 positive cells. (**B**) p-JAK2 positive cells. (**C**) STAT3 positive cells. (**D**) p-STAT3 positive cells.

Figure 5A–5D shows that the brown-yellow granular cells in the cytoplasm, nucleus, and membrane are JAK2-/p-JAK2-, STAT3-, and p-STAT3-positive cells, respectively. The immunohistochemical staining results show that the expressions of JAK2-, p-JAK2-, STAT3-, and p-STAT3-positive cells in the CIA group are significantly higher than that in the control group. With pharmacological intervention, the immunohistochemical sections show that the expressions of JAK2, p-JAK2, STAT3, and p-STAT3 significantly decrease in the WJHL and leflunomide groups and the effect of relief significantly enhances with the improvement of WJHL treatment. The medium-dose and high-dose WJHL groups exhibit better inhibitory effect on JAK2, p-JAK2, STAT3, and p-STAT3 protein expressions.

### Effect of WJHL on the proportion of Th17 cells in the spleen of CIA mice

**Figure 6:**
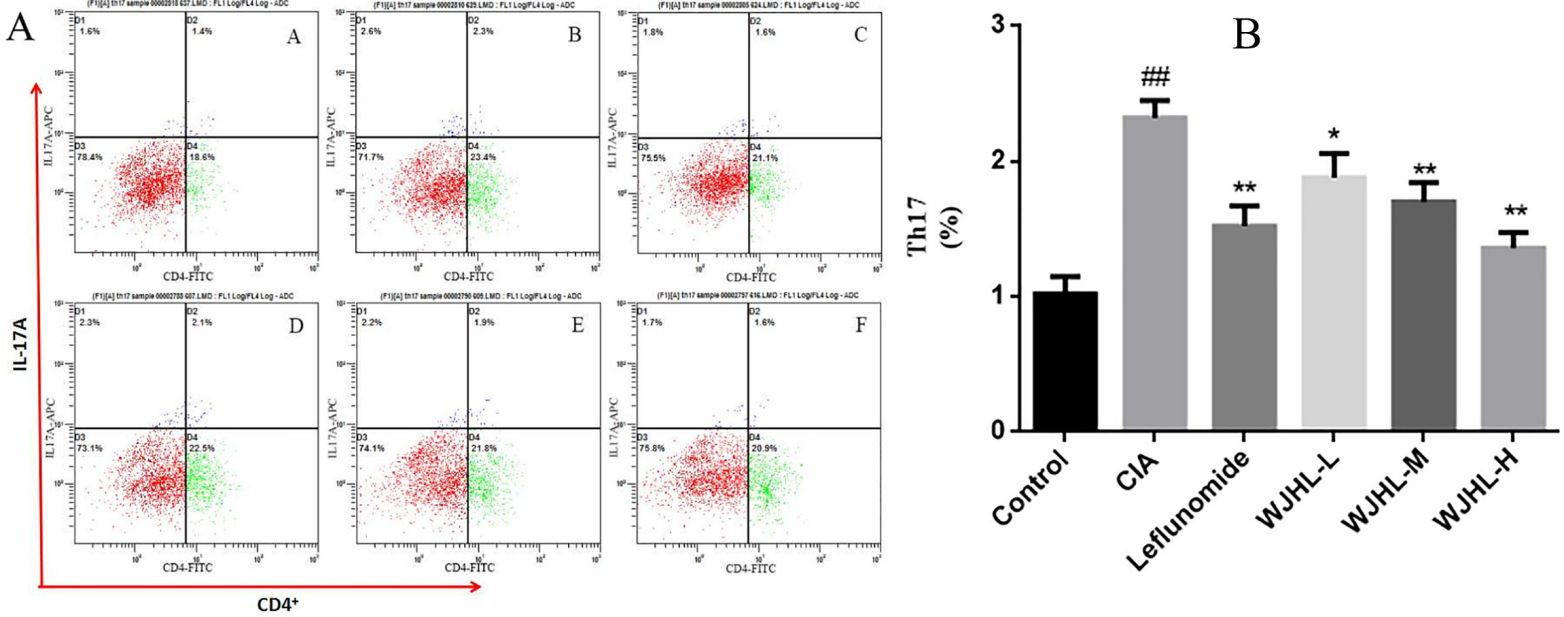
Effect of WJHL on the proportion of Th17 cells in the spleen of CIA mice Values are the mean ± SEM (*n* = 10). *P < 0.05, ^*^P < 0.01 compared with the CIA group; ^#^*P <* 0.05, ^##^*P <* 0.01 compared with the control group. (**A**) Flow cytometry of CD4^+^ T-IL-17A cells. (**B**) The proportion of CD4^+^ T-IL-17 cells.

CD4^+^ Th cells which include the Th17 cells are the major components of the adaptive immune response system and play an important role in the regulation of antigen-specific immune responses. IL-17A released by Th17 cells can promote CIA arthritis, and the inhibition of IL-17A can reduce arthritis. The percentage of CD4^+^ T-IL-17A cells is measured by flow cytometry, as shown in Figure 6A and 6B. The proportion of CD4^+^ T-IL-17 cells in the CIA group is significantly higher than that in the control group (*P < 0.01*). The proportions of CD4^+^ T-IL-17A cells in the leflunomide and WJHL groups (medium-dose and high-dose) are significantly lower than that in the CIA group (*P < 0.01*). The study shows that Th17 differentiation plays an important role in the pathogenesis of CIA mice. WJHL can relieve the arthritis by inhibiting Th17 activation and decreasing IL-17A secretion.

### Contents of inflammatory and anti-inflammatory factors in serum of CIA mice tested by enzyme-linked immunosorbent assay (ELISA)

**Figure 7:**
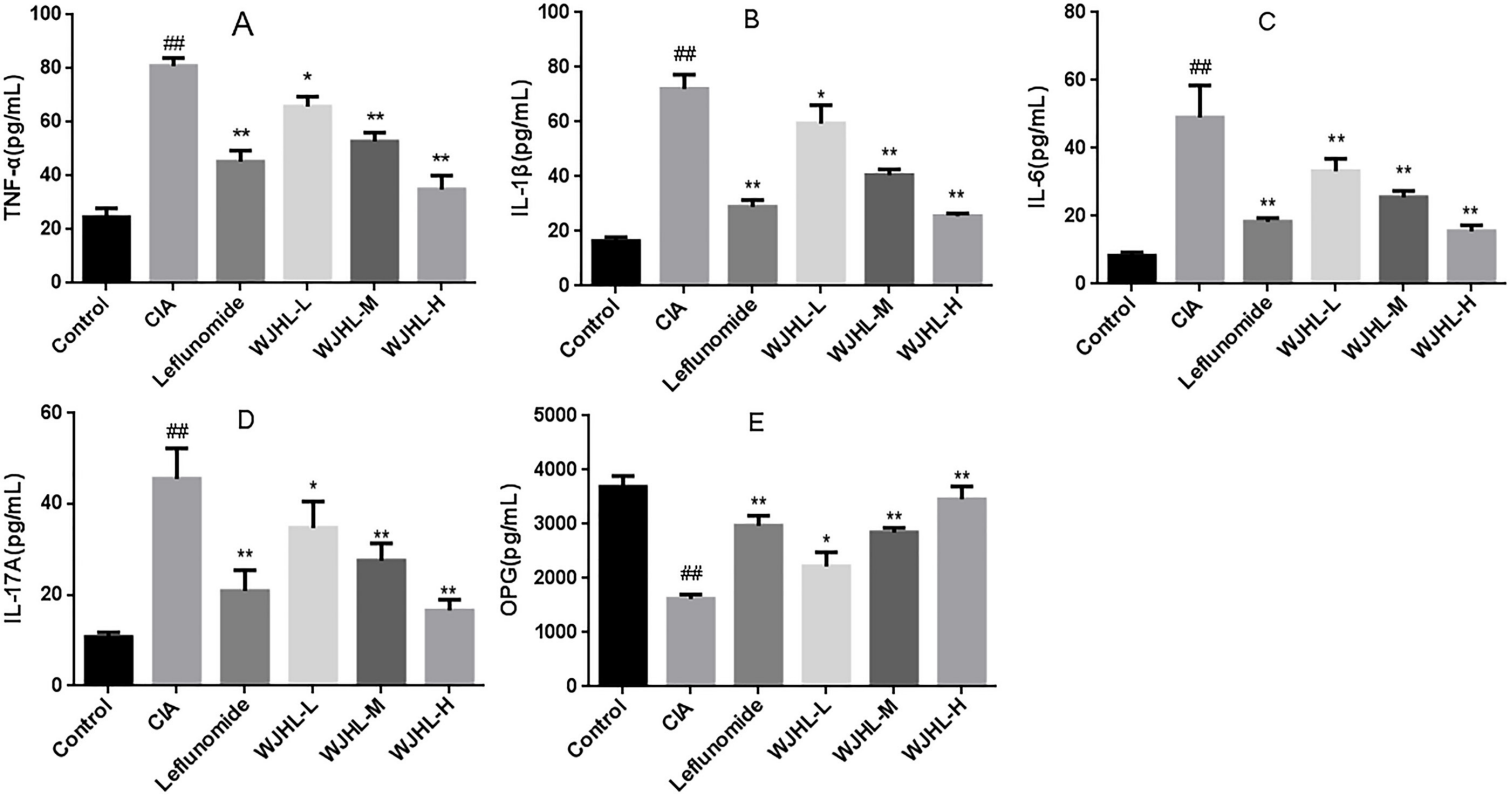
Contents of inflammatory and anti-inflammatory factors in serum of CIA mice Values are the mean ± SEM (*n* = 10). *P < 0.05, ^*^P < 0.01 compared with the CIA group; ^#^*P <* 0.05, ^##^*P <* 0.01 compared with the control group. (**A**) Contents of TNF-α. (**B**) Contents of IL-1β. (**C**) Contents of IL-6. (**D**) Contents of IL-17A. (**E**) Contents of OPG.

Figure 7A–7D shows that the contents of TNF-α, IL-1β, IL-6, and IL-17A in the serum of CIA mice in the CIA group increase significantly (*P < 0.01*), respectively, whereas Figure 7E indicates that the content of osteoprotegerin (OPG) decreases significantly (*P < 0.01*). This finding indicates that TNF-α, IL-1β, IL-6, IL-17A, and OPG play a role in the pathogenesis of CIA and that TNF-α, IL-1β, IL-6, and IL-17A play a synergistic role in the immune response and tissue damage of RA. The levels of TNF-α, IL-1β, IL-6, and IL-17A in the WJHL groups (medium-dose and high-dose) decrease significantly (*P < 0.01*), whereas that of OPG in the WJHL groups (medium-dose and high-dose) increases significantly (*P < 0.01*). The effects of the high-dose WJHL group are superior to those of the leflunomide group, which indicate that both of leflunomide and WJHL groups could decrease the levels of TNF-α, IL-1β, IL-6, and IL-17A and increase the level of OPG to treat RA. The mechanism may be to reduce the levels of cytokines, reduce joint local synovitis and cartilage damage, and inhibit synovial cells from secreting protease to reduce joint and cartilage damage to achieve the therapeutic purpose.

### Expressions of JAK2, p-JAK2, STAT3, and p-STAT3 in synovial tissues of CIA mice tested by Western blot

**Figure 8:**
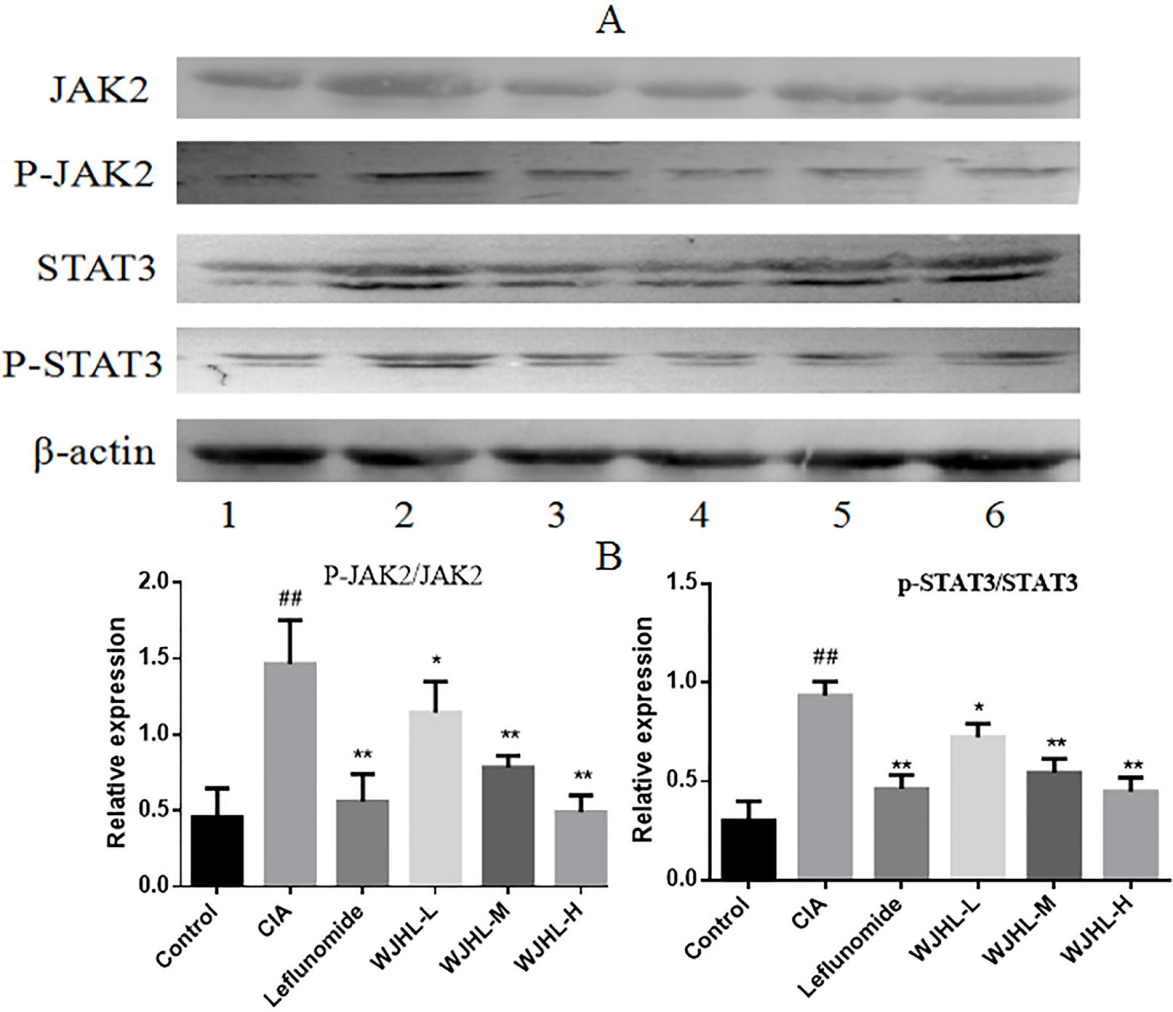
Protein expressions of JAK2, p-JAK2, STAT3, and p-STAT3 in synovial tissues of CIA mice Values are the mean ± SEM (*n* = 10). *P < 0.05, ^*^P < 0.01 compared with the CIA group; ^#^*P <* 0.05, ^##^*P <* 0.01 compared with the control group. (**A**) Representative western blot showing the protein expression of JAK2, p-JAK2, STAT3, and p-STAT3. 1: Normal group. 2: CIA group. 3: Leflunomide group. 4: High-dose of WJHL group. 5: Medium-dose of WJHL group. 6: Low-dose of WJHL group. (**B**) Relative intensity values from band densitometry of p-JAK2/JAK2 and p-STAT3/STAT3.

Figure 8A shows that the expressions of JAK2, p-JAK2, STAT3, and p-STAT3 are upregulated in the synovial tissue of the CIA group. A clear band of expression, which significantly increases compared with that in the control group, is observed. In the WJHL and leflunomide-positive groups, a band of expression for JAK2, p-JAK2, STAT3, and p-STAT3 is observed, but the expression levels are lower than that of the CIA group. The band of expression decreases along with the increase in dose. WJHL inhibits the protein expression of p-JAK2 and P-STAT3, and the effect becomes more obvious when the dose is increased.

Figure 8B shows that the relative expressions of p-JAK2/JAK2 and p-STAT3/STAT3 in synovial tissues of the CIA group are significantly higher than that of the control group (*P < 0.01*). The expressions of p-JAK2/JAK2 and p-STAT3/STAT3 are significantly inhibited in the WJHL (medium-dose and high-dose) and leflunomide groups compared with that in the CIA group (*P < 0.01*). The effect of the high-dose WJHL group is better than that of the leflunomide group.

### Relative expressions of JAK2, STAT3, IL-17A, RORγt, Foxp3, and OPG mRNA in synovial tissues of CIA mice

**Figure 9:**
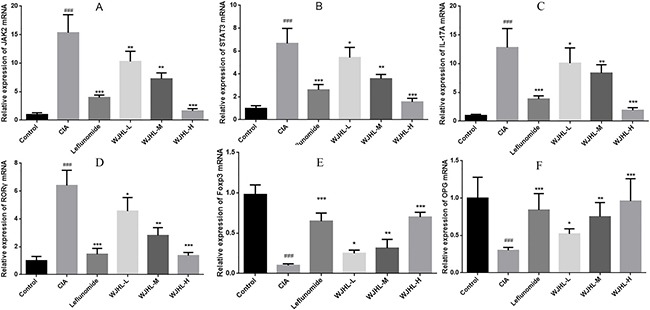
Relative expressions of JAK2, STAT3, IL-17A, RORγt, Foxp3, and OPG mRNA in synovial tissues of CIA mice Values are the mean ± SEM (*n* = 10). *P < 0.05, ^*^P < 0.01, and ^**^*P < 0.001 compared with the CIA group; ^#^*P <* 0.05, ^##^*P <* 0.01, and ^###^*P <* 0.001 compared with the control group. (**A**) JAK2 mRNA expressions (**B**) STAT3 mRNA expressions (**C**) IL-17A mRNA expressions (**D**) RORγt mRNA expressions (**E**) Foxp3 mRNA expressions (**F**) OPG mRNA expressions.

Figure 9A–9F shows the mRNA expressions of JAK2, STAT3, IL-17A, RORγt, Foxp3, and OPG in synovial tissues of CIA mice, respectively. Quantitative polymerase chain reaction (QPCR) analysis shows that, compared with the control group, the mRNA expression levels of JAK2, STAT3, IL-17A, and RORγt in the CIA group are significantly higher than that in the control group (*P < 0.001*), whereas the mRNA expression levels of Foxp3 and OPG significantly decrease (*P < 0.001*). Administration makes the mRNA expression levels of JAK2, STAT3, IL-17A, and RORγt decrease in leflunomide and WJHL groups, whereas the mRNA expression levels of Foxp3 and OPG significantly increase (*P < 0.05, P < 0.01, and P < 0.001*). The high-dose WJHL group is superior to the leflunomide-positive group.

### Effect of WJHL on the expressions of JAK2 mRNA, STAT3 mRNA, IL-17A mRNA, RORγt mRNA, Foxp3 mRNA, and SOCS3 mRNA in splenic CD4^+^ T cells of CIA mice

**Figure 10:**
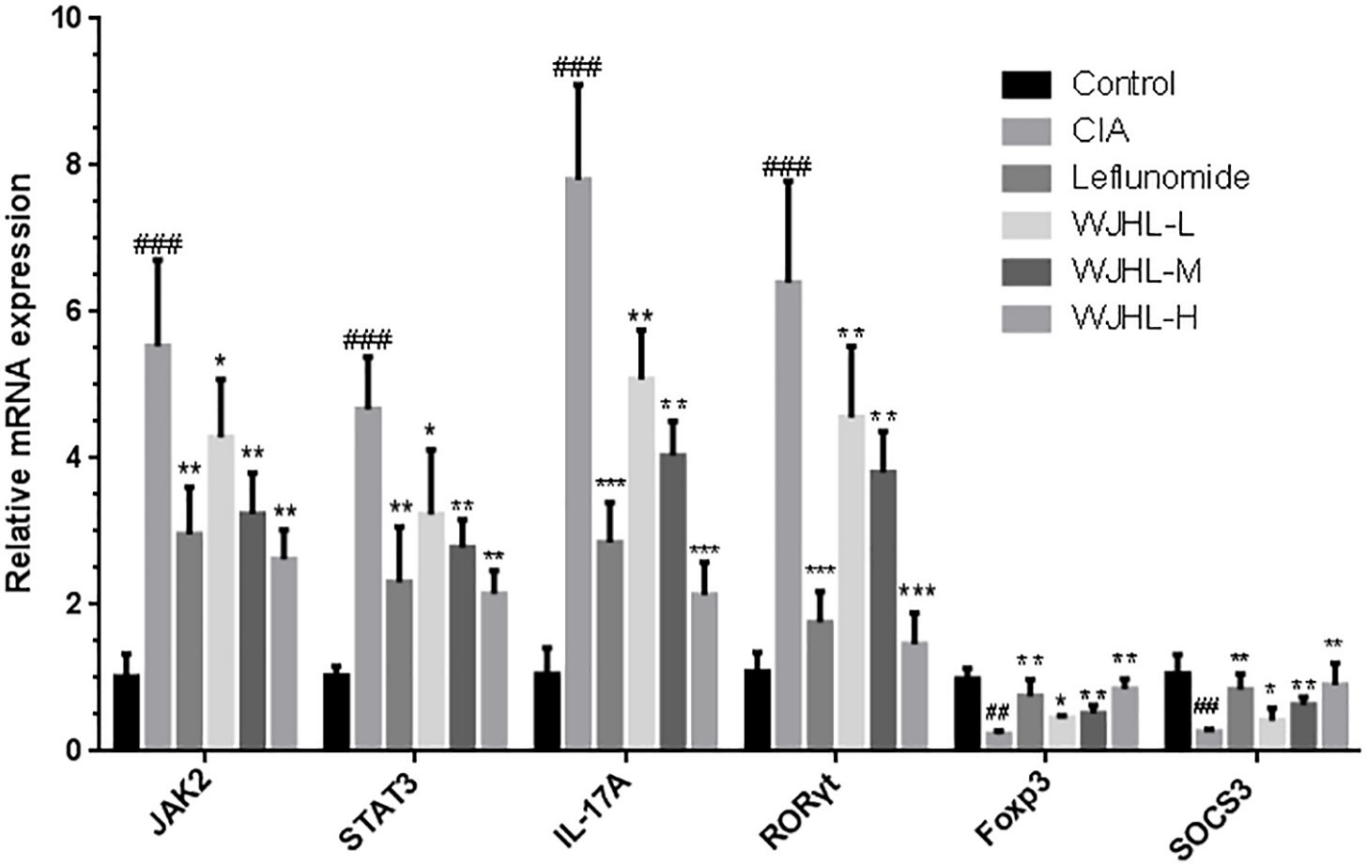
Effect of WJHL on the expressions of JAK2, STAT3, IL-17A, RORγt, Foxp3, and SOCS3 mRNA in splenic CD4^+^ T cells of CIA mice Values are the mean ± SEM (*n* = 10). *P < 0.05, ^*^P < 0.01, and ^**^*P < 0.001 compared with the CIA group; ^#^*P <* 0.05, ^##^*P <* 0.01, and ^###^*P <* 0.001 compared with the control group.

Th17 cell differentiation plays a key role in RA inflammation and bone destruction. T cell differentiation in inflammatory sites is mediated by JAK2/STAT3 signaling pathway activation. Figure 10 shows that the high expression of JAK2 in the CIA group is five times, STAT3 is four times, IL-17A is seven times, and RORγt is approximately six times higher than that in the control group, whereas these expressions of the WJHL groups significantly decrease compared with the CIA group, indicating a better inhibition and a statistical significance. The results show that WJHL could inhibit the expressions of JAK2, STAT3, IL-17A, and RORγt mRNA in splenic CD4^+^ T cells of CIA mice. Furthermore, Foxp3 and SOCS3 genes in the CIA group have more than fourfold low expression compared with the control group. However, the expression levels of Foxp3 and SOCS3 mRNA can be upregulated after the WJHL dose groups are given pharmacological intervention compared with the CIA group, indicating that WJHL could upregulate the expression levels of negative feedback modulator SOCS3 mRNA and regulatory T cells (Treg)-specific transcription factor Foxp3 mRNA, which can significantly change RA symptoms based on the increase or decrease in dose.

## DISCUSSION

The JAK/STAT signaling pathway is an important cytokine signal transduction pathway and is one of the common pathways of physiological and pathological reactions in the human body, playing an important role in the regulation of cell proliferation, differentiation, apoptosis, immune response, inflammation, cancer, and other pathophysiological processes. Of which, STATs tyrosine phosphorylation is the key link in the activation, dimerization, and DNA binding that regulate transcription factors to play a variety of biological effects. Modern studies show that synovial hyperplasia of RA is the pathological basis to cause joint damage. The JAK2/STAT3 pathway has been inherently linked with cell differentiation and proliferation and has become the important path for the study of the transfer of information and regulation of synovial cells. In this study, DBA/1 mice are used to set up a CIA animal model with considerable similarity to RA clinical manifestations and pathological changes. DBA/1 mice are also an animal model commonly used in the study of RA efficacy and mechanism. WJHL, characterized by monarch, minister, adjuvant and assistant emphasizes the integrated treatment based on multi-component action instead of the single-component therapy, which is the typical superiority of traditional Chinese medicine prescription. The results show that WJHL can significantly alleviate the progression of CIA, reduce ankle swelling, arthritis score, pathological changes, and bone destruction, and increase the level of OPG by inhibiting the levels of TNF-α, IL-1β, IL-6, and IL-17 in the serum of CIA mice. Real-time QPCR determined that WJHL could decrease the mRNA expressions of JAK2, STAT3, IL-17A, and RORγt at different levels and significantly increase the mRNA expressions of Foxp3, OPG, and SOCS3. WJHL could inhibit the generation and activity of these inflammatory cytokines and reduce RA-related symptoms to achieve the purpose of the treatment of RA. WJHL can inhibit the expressions of JAK2, p-JAK2, STAT3, and p-STAT3 and the relative expressions of p-JAK2/JAK2 and p-STAT3/STAT3 in synovial tissues and can regulate the JAK2/STAT3 pathway, which is consistent with the articles in the literature.

T and B cell immune dysfunction is one of the main mechanisms of RA disease, in which T cells play a central role. Th17 cells are another Th subpopulation different from the Th1 and Th2 cell differentiation pathways. IL-17 is a characteristic cytokine derived from Th17 cells, which is associated with inflammatory diseases. IL-17 can enhance the inflammatory response. IL-17 has been found in a variety of autoimmune diseases, such as RA and lupus erythematosus. IL-17, as a proinflammatory cytokine, is secreted by chondrocytes mediated by T cells and can regulate the activated DCs to secrete IL-1β, TNF-α, and IL-6. IL-17 may promote the expressions of STAT3 and RORγt and secrete IL-17. The levels of Th17 cells and IL-17 cytokines in CIA mice increase significantly, so the inhibition of the generation of Th17 cells and IL-17 cytokines can improve the symptoms of RA. Therefore, Th17 cells play an important role in the pathogenesis of RA.

In this study, splenic CD4^+^ T cells are isolated through immunomagnetic beads. The expressions of the transcription factors JAK2, STAT3, IL-17A mRNA, RORγt mRNA, Foxp3 mRNA, and SOCS3 mRNA in splenic CD4^+^ T cells are detected by fluorescence QPCR. RORγt and IL-17A mRNA represent the cellular level of Th17 cells, and Foxp3 mRNA represents the cellular level of Treg cells. The expression level of JAK2 and STAT3 mRNA represents the activation state of the signaling pathway of JAK/STAT in CD4^+^ lymphocytes. The results show that the WJHL groups can significantly inhibit the expressions of JAK2, STAT3, IL-17A, and RORγt mRNA, obviously increase the expressions of Foxp3 and SOCS3 mRNA, and significantly decrease the proportion of CD4^+^ T–IL-17A cells in the spleen.

## MATERIALS AND METHODS

### CIA model construction and experimental grouping of DBA/1 mice

A total of 60 healthy and clean male DBA/1 mice, weighing approximately 18 g to 20 g, were provided by the Laboratory Animal Research Center, Zhejiang Chinese Medical University. The mice were kept under natural light, with six mice each cage. Food and water were freely available, the room temperature was kept at 25 ± 3°C, and the ventilation was good. Each group of DBA/1 mice was fed adaptively for one week and starved 1 day before the experiment.

A certain amount of collagen type II (collagen II, CII) was dissolved in 0.1 M acetic acid at a concentration of 2 mg mL^−1^. The mixture was stirred at 4°C and kept in a refrigerator at 4°C overnight. The mixture was emulsified in 1:1 mixture in an ice water bath to prepare a CII emulsion (i.e., a CII concentration of 1 mg mL^−1^) and stored in a refrigerator at 4°C. When modeling was done, each mouse was injected with 0.1 mL of CII emulsion at the end of the tail, and another 0.1 mL was injected at the end of the tail on the 7th day, and the CIA model was evaluated.

Six DBA/1 mice were randomly selected as the control group. CIA mice were randomly divided into CIA group, leflunomide group, low-dose WJHL group, medium-dose WJHL group, and high-dose WJHL group, with six mice in each group. WJHL-lyophilized powder was dissolved in 0.5% carboxymethyl cellulose natrium. From the 30th day of primary immunization, the WJHL group was given WJHL decoction for 1.25, 2.50 and 5.00 g/kg. The positive group was given leflunomide (6 mg/kg/d), and the control and CIA groups were given 0.2 mL/10 g saline per day. The animals were sacrificed on the 69th day of *administration* and their sera were taken for study. The blood was centrifuged and frozen at −80 °C. Tissues of the right posterior ankle synovium, spleen, and thymus were taken and placed in 10% paraformaldehyde to be measured.

### Preparation of WJHL-lyophilized powder

A certain amount of qualified decoction pieces of *C. cassia* Presl., *Cinnamomum cassia* Presl., *Paeonia lactiflora* Pall.*, Saposhnikovia divaricate* (Turcz.) Schischk., *and Clematis chinensis* Osbeck was weighed. Then, 10 times deionized water was added to soak for 30 min and decocted for 2 h. The decoction was poured and filtered. Then, eight times deionized water was added to the residue to decoct for 1.5 h, mixed with previously obtained decoction, and filtered with five layers of gauze to obtain a concentrated decoction. The decoction was freeze-dried through the freeze-drying technique and quickly placed in a sealed bag for preservation. The yield was approximately 25% (that is, 100 g herbs yield 25 g freeze-dried powder).

### Histopathological examination (H&E staining)

Mice were sacrificed, and the ankle joint, knee joint, and their surrounding tissues were fixed with 10% formaldehyde for 12 h, dissolved in 2,000 mL PBS with 200 g 10% EDTA-2Na, and adjusted to pH 7.2–7.4 with NaOH (4 μm). Decalcification treatment was conducted twice a week. Considerable work should be done, including decalcification for two months, gradient ethanol dehydration at different concentrations, sectioning of paraffin-embedded tissue (4 μm), and H&E staining. Pathological changes of the ankle of mice were observed under a microscope and images were taken. Changes of the synovial lesions, inflammatory cell infiltration, pannus hyperplasia, bone and cartilage injury, and other pathological changes were also observed. The specific scoring criteria were as follows: 0, normal; 1, mild inflammatory synovial cell infiltration; 2, moderate inflammatory cell infiltration accompanied by early cartilage loss; 3, severe inflammation, pannus formation, significant cartilage loss, and bone erosion around the joints.

### X-ray examination and MICRO-CT detection of the posterior ankle of mice

On the 69th day, images of the ankle and foot joints of mice were taken by means of the small animal biopsy imaging technique. Then, imaging examination and analysis were conducted. The swelling degree of joint soft tissue and the destruction of joint bone were observed before and after CIA mice were administrated with *drugs*.

On the 69th day of the experiment, the entire tibia and feet of each group were surgically removed. The skin and surface muscle were stripped carefully, and every specimen was labeled with the time. The tissue samples were soaked and fixed with 6% paraformaldehyde. Seven days later, the specimen was placed and scanned in the MICRO-CT machine. GE's three-dimensional data analysis software MicroView ABA version 2.1.2 was used to make a three-dimensional reconstruction of the ankles, describe the bone morphology, and directly observe the efficacy of WJHL on the CIA mice.

### Expressions of JAK2, p-JAK2, STAT3, and p-STAT3 in spleen tissue tested by immunohistochemical staining

Sections of spleen tissues were prepared. Then, the HPIAS-2000 high-resolution color computer image analysis system was used to analyze the results of immunohistochemical staining. A total of 10 visual fields were selected randomly for each group. A microscope was used to magnify the tissue sections by 200 times, capture the image based on a 512 × 512 pixel distribution, and save the image in the computer image analysis system. An immunohistochemical analysis model was used to analyze the two groups of sections. The images were segmented. The point-like segmentation was selected to detect positive cell absorbance, and the percentage of positive cells was analyzed to account for the total area of the cells. The product represents the relative content of the antigen in the tissues, and the larger the product is, the higher the antigen content. In the same ploidy and background, each group makes an automatic quantitative analysis and records the results.

### Effect of WJHL on the proportion of Th17 cells in the CIA mice model

On the 69th day, CIA mice were sacrificed, and the CD4^+^ T cell isolation kit (containing biotin-labeled antibody cocktail and biotin-labeled magnetic beads) was used to separate CD4^+^ T cells. The adjusted cell count was 5 × 10^7^/mL. Then, the cells were resuspended in a 5 g/L BSA and 2 mol/L EDTA sorting buffer. The appropriate amount of biotin-labeled cocktail antibody (including anti-mouse CD8, CD14, CD16, CD19, CD36, CD56, CD123, TCR, and blood type glycoprotein A) was added and mixed. The cells were incubated for 10 min at 4°C to 8°C. Anti-Biotin microbeads were added, mixed, and incubated for 15 min at 4°C to 8°C. Then, the cells were washed in buffer and centrifuged at 1,000 rpm for 10 min. The liquid supernatant was removed to obtain the sediment, which was resuspended in 500 μL buffer. The LD column was placed in a MidiMACS classifier, and 2 mL buffer was poured into the column. The cell suspension was added, and the cells that flowed out of the column were obtained.

### The levels of TNF-α, IL-1β, IL-6, IL-17A, and OPG in the serum of mice measured by ELISA

On the 30th day (before treatment) and the 90th day (after treatment) since the model was set up, blood was taken from the posterior venous plexus of each group of mice, and serum was taken after the blood has coagulated and centrifuged at 2,500 rpm for 5 min. The ELISA kit was used to detect the expression levels of cytokines TNF-α, IL-1β, IL-17A, IL-6, and OPG in serum.

### Detection of protein expression by western blot

Whole-cell lysates were prepared using radioimmunoprecipitation analysis buffer supplemented with protease and/or phosphatase inhibitors. The protein levels were determined using a BCA assay kit (Pierce, USA). Proteins (50 μg/well) were separated by SDS–polyacrylamide gel electrophoresis, transferred to a PVDF membrane (Millipore, Burlington, MA, USA), and blocked with 5% skim milk in Tris-buffered saline containing 0.1% Tween 20. Target proteins were detected by corresponding primary antibodies, and subsequently by horseradish peroxidase-conjugated secondary antibodies. Protein bands were visualized using chemiluminescence reagent (Millipore, Burlington, MA, USA). Equivalent loading was confirmed using an antibody against β-actin. The levels of target protein bands were densitometrically determined using Quantity Ones 4.4.1 (Bio-Rad Laboratories, Berkeley, CA, USA). The variation in the density of bands was expressed as fold changes compared to that of control in the blot after normalization to β-actin or the total protein in some experiments.

### Detection of gene expression by QPCR

Total RNA was extracted and converted to cDNA using the RevertAidTM First Strand cDNA synthesis kit following the supplier's instructions. β-actin was used as a control for adjusting the relative of total RNA. The experiment was carried out by two-step PCR standard procedure (pre-denaturation: 95°C for 10 min, PCR reaction: 95°C for 15 s and 68°C for 60 s for 40 cycles). The PCR reactions for JAK2, STAT3, IL-17A, RORγt, Foxp3, OPG, and SOCS3 were analyzed by 2% agarose gel electrophoresis. Real-time fluorescent quantitative PCR amplification was carried by using ABI7500 Fast Real-Time PCR System at least three times.

### Statistical analysis

The results are presented as means ± SEM. Data were analyzed by one-way ANOVA. Comparisons between two groups were performed using the Dunnett's multiple comparisons test or post-hoc analysis. Statistical analyses were carried out using GraphPad Prism version 6.0 (GraphPad Software, San Diego, CA, USA). *P < 0.05* was considered statistically significant. *P < 0.01* was considered higher statistically significant, and *P < 0.001* was considered the highest statistically significant.

## Abbreviations

RA: Rheumatoid arthritis
CIA: collagen-induced arthritis
OPG: osteoprotegerin
DCs: dendritic cells
APCs: antigen-presenting cells
JAK: janus tyrosine kinase
STAT: signal transducer and activator of transcription
WJHL: wenjinghuoluo prescription
